# Data-driven derivation of macroscopisc fundamental diagram from floating car trajectories

**DOI:** 10.1371/journal.pone.0342070

**Published:** 2026-02-03

**Authors:** Xiaojuan Lu, Jiamei Zhang, Qingling He, Shiyu Zheng, Juan Su

**Affiliations:** 1 School of Traffic and Transportation, Lanzhou Jiaotong University, Lanzhou, China; 2 College of Transportation Engineering, Chang’an University, Xi’an, China; 3 School of Economics and Management, Qingdao Institute of Technology, Qingdao, China; Southwest Jiaotong University, CHINA

## Abstract

This study proposes a novel GPS-based methodology for Macroscopic Fundamental Diagram (MFD) estimation to overcome limitations of fixed detectors and inaccurate penetration rate assumptions. The approach dynamically identifies stop-line positions using spatiotemporal floating car data, calculates maximum queue lengths per signal cycle by combining floating car positions with estimated arriving vehicle lengths, and establishes a speed-based nonlinear model to determine queuing vehicle counts. A dynamic scaling coefficient derived from maximum queue lengths enables assumption-free estimation of total regional vehicles when applied to the floating car population. Validation using Chengdu data demonstrates significant improvements: unary cubic curves achieve optimal fitting for MFD relationships (R^2^ up to 0.9157); the HMM-CRF hybrid map-matching algorithm reduces average position error by 29% and intersection mismatch rate by approximately 40%; simulation results show queue length estimation accuracy of RMSE 22.8m and MAPE 18.5%, while MFD estimation error for maximum network flow drops from −17.5% to −3.5%, representing an 80% relative accuracy improvement. The proposed methodology provides robust technical support for urban road network assessment and management by enabling high-precision acquisition of MFDs from floating car data, effectively addressing critical challenges in macroscopic traffic modeling and monitoring. This advancement presents potential value for perimeter control applications and other MFD-based traffic management strategies.

## 1 Introduction

In recent years, advancements in intelligent traffic data collection technologies have led to significant improvements in obtaining the Macroscopic Fundamental Diagram (MFD). The MFD captures the patterns of variation in network-averaged traffic parameters, including flow, density, and speed. By developing traffic flow models that exhibit unimodal stability, optimal traffic flow control can be effectively achieved.

**Fig 1:**
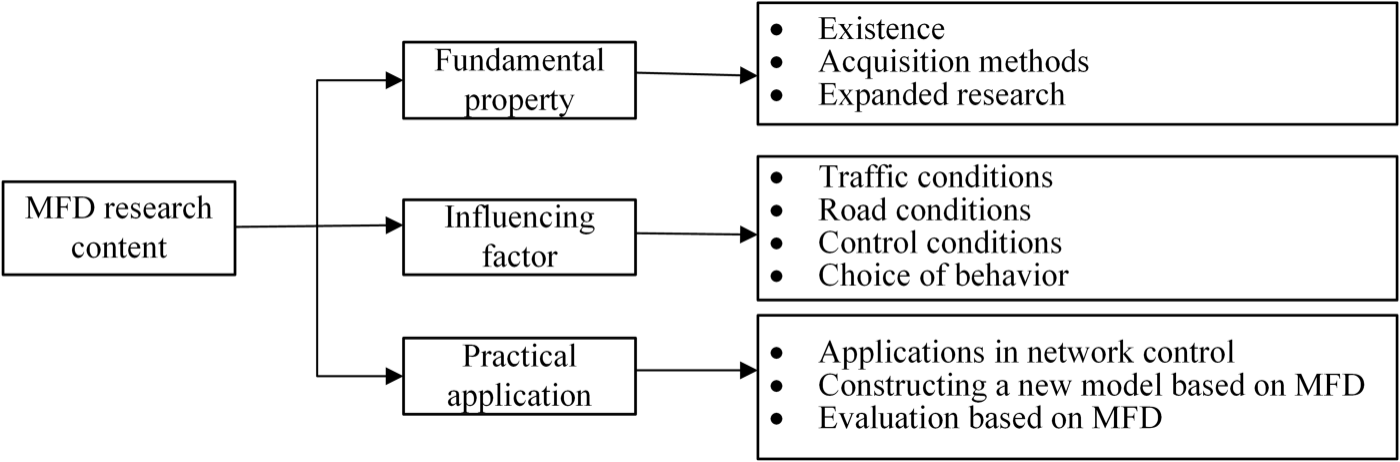
Main research context of MFD.

The MFD, also known as the Network Fundamental Diagram (NFD) or Arterial Fundamental Diagram (AFD) in some studies, has undergone significant exploration [1]. In 1969, Godfrey [2] was a pioneer in examining the relationship between network-averaged speed and density using floating car data. His findings revealed that the total vehicle distance traveled within a network peaks within specific density ranges, setting the stage for future research. In 2007, Daganzo [3] further derived a speed-density relationship diagram through microscopic simulation experiments in San Francisco’s commercial district, suggesting the potential existence of MFDs in homogeneous areas. In 2008, Geroliminis and Daganzo [4] employed real-world data from Yokohama, Japan, to validate the existence of MFDs in large-scale urban networks, connecting spatially averaged flow, density, and speed. They concluded that the MFD is an inherent characteristic of road networks. These studies have garnered considerable attention from transportation experts and scholars worldwide, laying the foundation for MFD applications in traffic control and network analysis. For detailed reviews on verifying MFD existence, studying heterogeneity and hysteresis effects, and estimating MFDs with limited data, please refer to the relevant literature [5]. The primary research framework is depicted in Fig 1.

The MFD of urban transportation networks plays a pivotal role in understanding and controlling traffic flow within urban road systems. Its essence lies in unveiling the macroscopic properties of traffic networks, particularly in homogeneous regions with uniformly distributed traffic density. The MFD demonstrates a unimodal, low-scatter relationship between the cumulative number of vehicles and network throughput within a specific area. Currently, two primary methods are employed to obtain MFD:

(1) Simulation-Based Modeling

Traffic simulation software, such as VISSIM and SUMO, is prevalent in this context, while some researchers have also modeled and calibrated MFD using Aimsun [6]. These simulation-based approaches offer a thorough exploration of network MFDs, providing vital data and insights for optimizing and understanding traffic flow [7].

(2) Empirical Data-Based Modeling

Two common techniques for collecting real-world traffic information are fixed loop detector data (LDD) and floating car data (FCD). LDD utilizes sensors to estimate traffic conditions (e.g., flow, speed) at specific locations, while FCD relies on GPS devices to gather vehicle trajectory data, including position and speed [8]. Both data sources facilitate real-time collection, enabling dynamic estimation of network MFDs. Notably, in 2008, Geroliminis and Daganzo [4] first validated the existence of MFDs using LDD and FCD from a 10 km^2^ road network in Yokohama, Japan, thereby establishing a cornerstone for subsequent research endeavors.

LDD has been extensively utilized in MFD studies [9]. Shi et al. [10] validated the MFD of Shanghai’s expressway network using LDD, attributing hysteresis effects to uneven traffic distribution. Alonso et al. [11] correlated LDD-measured flow and density with signal control plans across different weekday periods. Paipuri et al. [12] fused location-based services (LBS) data, OD matrices, and LDD to estimate time-varying penetration rates for multi-region MFD simulations. Mariotte et al. [13] compared simulated MFD-predicted flow (total accumulation and average speed) with real-world data from loop detectors and probe sensors in Lyon, France. Ambühl et al. [14] utilized one year of fixed traffic sensor data from Zurich and Lucerne, employing MFD concepts and dynamic time warping (DTW) algorithms. Lee et al. [15] investigated biases in loop detector positioning on MFDs using analytical and simulation methods alongside empirical data from UTD19. Maiti et al. [16] proposed two alternative approaches: (1) hierarchical network scaling and (2) variogram-based data imputation. The results demonstrate that the hierarchical scaling method achieves the most accurate estimates, maintaining reliable performance with only 5% uniform detector coverage, whereas the variogram model fails to provide valid estimates. Building on this, Mousavizadeh et al. [17] further investigated how to simultaneously account for the combined effects of topological features and traffic flow characteristics to overcome the limitations of existing loop detector-based NMFD estimation methods.

Given budget constraints for detector equipment, installing detectors on every part of the road network is impractical, rendering road sections without detectors unmonitorable. Furthermore, fixed detectors are susceptible to random failures stemming from hardware or software malfunctions, network communication disruptions, power outages, and facility maintenance issues. Consequently, even on road sections equipped with fixed detectors, some traffic data may still be lost. To address these challenges, researchers have increasingly turned to utilize FCD to derive the MFD. Nagle et al. [18] proposed that when floating cars are evenly distributed across the road network, the weighted traffic flow and density of the network can be estimated using the known penetration rate of these floating cars. Consequently, the challenge of determining the penetration rate of floating cars has garnered considerable attention from scholars. Lu et al. [19] introduced the Two-Fluid Model (TFM) for urban traffic and estimated the taxi penetration rate by calibrating real GPS data from Changsha City to obtain the MFD for extensive urban areas. Saffari et al. [20] presented a methodology to estimate the MFD for large-scale urban networks solely using probe vehicle trajectories, without needing prior knowledge of the penetration rate. Additionally, Shim et al. [21] examined the bifurcation phenomenon that arises in MFD estimation when relying on roadside detectors.

The challenge of utilizing FCD to derive the MFD lies in the potential accuracy issues caused by low penetration rates of floating cars. To enhance the precision of MFD estimation, researchers have explored the integration of FCD with LDD. Saffari et al. [22] utilized both LDD and FCD datasets, comparing fused data results with a baseline method relying solely on loop detectors, thereby validating the inherent limitations of loop detectors in measuring average density. In 2016, Ambühl et al. [23] introduced a method that concurrently utilizes LDD and FCD, fusing them to estimate the MFD. They validated the effectiveness of their fusion algorithm on both an abstract grid network and in the center of Zurich city. In 2017, Ambühl et al. [24] further demonstrated that the fusion of LDD and FCD outperforms MFD estimation using either data source individually. Lin et al. [25] proposed a data fusion model employing a backpropagation neural network, leveraging LDD and FCD to estimate network MFD parameters. Alonso et al. [26] also combined FCD with traffic data from traditional loop detectors to calibrate the MFD. Similarly, Maiti et al. [27] significantly improved the accuracy of LDD-based NMFD estimation by reconstructing average speed from loop detector data using FCD during the training phase.

A summary of empirical studies on MFD is presented in Table 1.

**Table 1:** Empirical studies of MFD.

Author	Literature	City	Data	Mode	Observed Traffic Conditions or Phenomena				
					Free Flow	Capacity	Congestion	Hysteresis	Bifurcation
Geroliminis et al. (2008)	[4]	Yokohama	LDD、FCD	Cars	√	√	√	×	×
Tilg (2020)	[9]	Munich	LDD	Cars	√	√	√	√	×
Shi et al. (2017)	[10]	Shanghai	LDD	Cars	√	√	√	√	×
Alonso et al. (2019)	[11]	Santander	LDD	Cars	√	√	×	×	×
Paipuri et al. (2020)	[12]	Dallas	LBS、LDD	Cars	√	√	√	×	×
Mariotte et al. (2020)	[13]	Lyon	LDD	Cars	√	√	√	×	×
Ambuihl et al. (2021)	[14]	Zurich & Lucerne	LDD	Cars	√	√	√	√	×
Maiti et al. (2025)	[16]	Barcelona	LDD, FCD	Cars	√	×	×	√	×
Mousavizadeh et al. (2024)	[17]	Athens	LDD	Cars	√	×	×	×	×
Lu et al. (2018)	[18]	Changsha	FCD	Cars	√	√	√	×	×
Shim et al. (2019)	[21]	Daegu	FCD	Cars	√	√	×	×	√
Saffari et al. (2022)	[22]	New Zealand	LDD	Cars	√	×	×	×	×
Ambühl et al. (2016)	[23]	Zurich	LDD、FCD	Cars	√	√	×	√	√
Ambühl et al. (2017)	[24]	Zurich	LDD、FCD	Cars	√	√	×	√	√
Alonso et al. (2023)	[26]	Santander	LDD、FCD	Cars	√	√	√	×	×
Maiti et al. (2025)	[27]	Athens, Lyon	LDD, FCD	Cars	√	×	×	×	×
Loder et al. (2017)	[28]	Zurich	LDD、FCD	Cars, Buses	√	√	√	×	×
Huang et al. (2019)	[29]	Shenzhen	FCD	Cars, Buses	√	√	×	√	√
Fu et al. (2020)	[30]	Shenzhen	LDD、FCD	Cars, Buses	√	√	×	√	×
Huang et al. (2022)	[31]	Shanghai	LDD、FCD	Cars, Bicycles	√	√	√	×	×
Tang et al. (2024)	[32]	Zurich	LDD	Cars, Buses	√	√	×	√	×
Chen et al. (2022)	[33]	Shenzhen	LDD、FCD	Cars, Buses	√	√	√	×	×

In summary, methods for obtaining the MFD through both simulation and measured data have matured and are widely used. Among these, the fusion of fixed LDD and FCD has proven effective in improving the accuracy of parameter estimation. Specifically, LDD is used to estimate the average density of the road network, while FCD is used to estimate the weighted flow. However, due to resource constraints, LDD is often limited in its ability to comprehensively cover large-scale road networks, whereas FCD is relatively easier to obtain. The penetration rate of floating cars is crucial for estimating the weighted traffic flow and density of the road network. In real-world scenarios, directly obtaining the total number of vehicles on the road network at a given time is challenging, often requiring estimation methods that may introduce errors, especially with incomplete or limited FCD. To address this, this paper introduces a method for estimating the maximum queue length using GPS data. By calculating the number of cars in a queue on a road segment within a specific time period, the proportion coefficient between cars and floating cars in the queue can be determined. This approach ultimately enables the acquisition of the MFD for the study area.

The key contributions of this article are as follows:

- Breakthrough in Sparse Data Utilization: We present the complete framework for constructing MFDs using only sparse floating car data, overcoming the traditional requirement for high penetration rates through dynamic vehicle proportion calibration.
- Real-Time Intersection Analytics: Our novel trajectory analysis method enables real-time quantification of signalized intersection queue lengths, particularly during critical red-light phases.
- Theoretical Advancement: We establish cubic polynomial curves as the optimal fitting model for fundamental MFD relationships (density-speed, density-flow), potentially useful for perimeter-control applications.

The paper is organized as follows: Section 2 presents the estimation of maximum queue length. Section 3 details the estimation of queued vehicle numbers. Section 4 describes the acquisition of the macroscopic fundamental diagram. A case study analysis is provided in Section 5. Finally, Section 6 concludes the paper and outlines future research directions.

## 2 Estimation of maximum queue length

Existing methods for estimating queue length predominantly rely on loop detector or video detection data, but these approaches come with inherent limitations. Device stability can compromise accuracy, loop detectors are prone to damage, and video detection systems often entail high costs. On the other hand, traditional GPS data was historically sourced from GPS devices installed in vehicles. However, advancements in technology have significantly refined GPS capabilities, enhancing the performance of GPS receivers to provide more precise and reliable location information. The GPS-based queue length estimation method capitalizes on this technological evolution by utilizing the position and time data of floating cars. It estimates the distance of each floating car to the stop line and, by multiplying the arrival rate during the residual red time by the duration of this period, calculates the newly accumulated queue length. This, in turn, enables the estimation of the total queue length of vehicles within a traffic signal cycle.

### 2.1 Estimation of stop line position

The accurate estimation of the stop line position is pivotal for precise queue length estimation. To enhance the accuracy of floating car data-based estimation, incorporating vehicle speed information serves as a valuable criterion. As vehicles draw nearer to the intersection, their speeds diminish considerably. Consequently, in proximity to the stop line, the concentration of slow-moving vehicles peaks. To estimate the location of the stop line using the low-speed critical threshold $V_{th}$ as the benchmark.

(1) Filter floating car data with speeds below the preset threshold $V_{th}$: Data with speeds below the preset threshold often correspond to situations where vehicles are nearly stopped or moving slowly, which can roughly determine the location range of the intersection where the stop line is situated.
(2) Divide the range and count the number of floating cars: To identify the area with the highest vehicle density, the filtered data range is divided into segments of δ meters, and the number of floating cars in each segment $f_{k}(k=1,2,3,\ldots ,\;\frac{D_{2}-D_{1}}{\delta })$ is counted, where k represents the segment index.
(3) Calculate the density for each segment: The density $\rho_{k}=\frac{f_{k}}{\delta }$ is obtained by dividing the number of floating cars in each segment by the length of the segment.
(4) Identify the position with the maximum density: The spatial range covered by the maximum density position $\rho_{\max}$ is $\rho_{\max}$, and the midpoint of this segment is taken as the location $\rho_{\max}$ of the stop line.

### 2.2 Estimation of the position of the last floating car in the queue

Based on the known traffic signal timing plan at road intersections, this paper conducts a relevant analysis of floating car data. First, during the red phase of the signal cycle, vehicles with a travel speed below the preset threshold $V_{th}$ are identified as queuing vehicles. Secondly, based on the positions of these queued vehicles, the distance between each vehicle and the stop line is calculated, and the results are ordered accordingly. By doing so, the farthest position from the stop line among these queued vehicles can be determined, which represents the position of the last floating car in the queue.

Generally, the data categories of floating cars primarily include time (t), ID identification number (id), latitude and longitude (x, *y*), and travel speed (v). In this paper, these attributes of floating cars are denoted by P(t,id,x,y,v), and the estimated position of the stop line is denoted by. (*X*, *Y*) Then, the distance L between the stop line and the position of the floating car is represented as:


$$L=\sqrt{{\left(x-X\right)}^{2}+{\left(y-Y\right)}^{2}}$$ (1)

**Fig 2:**
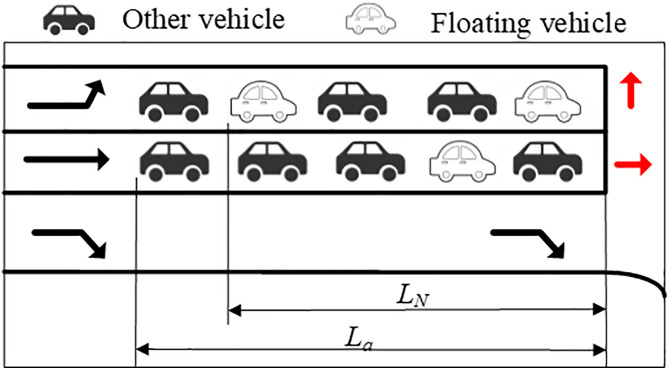
Schematic diagram of queuing scene.

As shown in Fig 2, the maximum queue length *L*_*a*_ at the intersection is jointly determined by the queue length $L_{N}$ of the floating car at the end of the queue and the length of the vehicle at the end of the queue.

**Fig 3:**
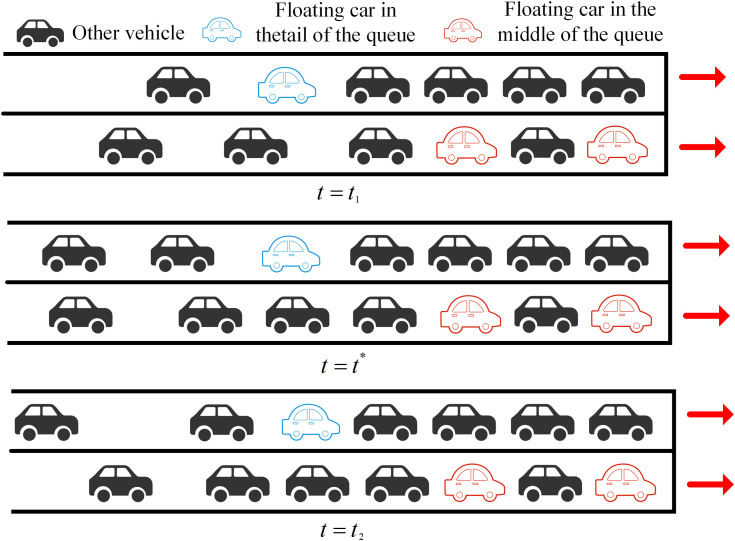
Schematic diagram of the parking process of a team-tailed floating vehicle.

Periodically transmitted floating car data reflect the current queuing status of vehicles, not their impending queue formation. In this paper, the state of a floating car that is about to enter the queue is designated as $P_{1}\left(t_{1},id_{1},x_{1},y_{1},v_{1}\right)$, and the state of being in the queue is designated as $P_{2}\left(t_{2},id_{2},x_{2},y_{2},v_{2}\right)$. Taking the blue floating car at the end of the queue in Fig 3 as an example, at time $t=t_{1}$, it is approaching the road intersection with other vehicles following behind; at time $t=t^{*}$. it begins to queue up while other vehicles continue to follow; and at time $t=t_{2}$, it is already in the queuing state. During the red phase of the signal cycle, the maximum queue length within the signal cycle can be determined by the sum of the queue length of the floating cars and the subsequently added queue length. Here, we introduce two concepts: “remaining red time,” which is the time from when the floating car at the end of the queue starts queuing until the red light ends; and “entry point,” which is the moment when a vehicle just enters the queue. The remaining red time can be derived from the entry point. As mentioned earlier, $P_{1}\left(t_{1},id_{1},x_{1},y_{1},v_{1}\right)$ and $P_{2}\left(t_{2},id_{2},x_{2},y_{2},v_{2}\right)$ can be obtained.

**Fig 4:**
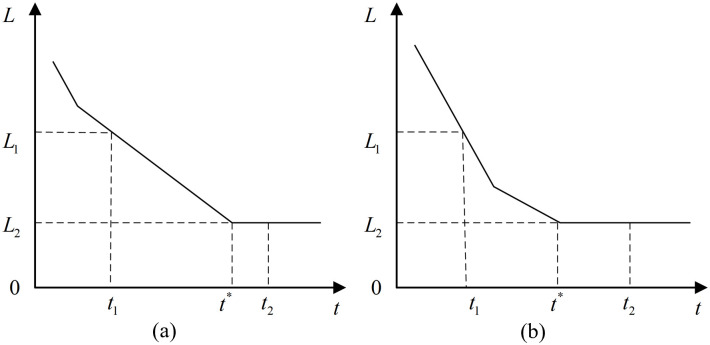
Vehicle distance from the stop line vs. time: (a) $v_{1}<v$; (b) $v_{1}\geq v$.


After calculating the distance between the vehicle and the stop line location, the pre-queue and post-queue data are denoted as $P_{1}\left(t_{1},id_{1},L_{1},v_{1}\right)$ and $P_{2}\left(t_{2},id_{2},L_{2},v_{2}\right)$, respectively. Based on the vehicle’s speed, its travel state can be determined. In real-world scenarios, vehicles typically travel at varying speeds. Thus, a critical speed $v^{*}$ is assumed: when a vehicle approaches an intersection with a speed greater than or equal to $v^{*}$, it continues to travel at a constant speed; when its speed falls below $v^{*}$, the vehicle decelerates uniformly until it joins the queue. Fig 4 illustrates the relationship between the vehicle’s distance to the intersection and time under these two distinct conditions. The moment $t^{*}$ at which the vehicle just joins the queue can be calculated as:

$$t^{*}=\left\{ \begin{array}{c} t_{1}+\frac{2\times \left(L_{1}-L_{2}\right)}{v_{1}},v_{1}<v^{*}\\ t_{1}+\frac{v_{1}}{a}+\frac{L_{1}-L_{2}-\frac{s_{1}^{2}}{2a}}{v_{1}},v_{1}\geq v^{*} \end{array}\right.$$ (2)

where a represents the vehicle’s deceleration; $L_{1}$ denotes the distance between the stop line and the floating car’s position before queuing; $L_{2}$ denotes the distance between the stop line and the floating car’s position after queuing; $v^{*}$ is the critical speed for uniform deceleration; $t_{1}$ represents the time before queuing.

As a pivotal traffic flow parameter, the arrival rate during the remaining red light period quantifies the arrival of vehicles between the tail floating car and subsequent downstream vehicles after the red light has been activated and until it concludes. The resultant new queue length is derived by multiplying the arrival rate by the duration of the remaining red light. Depending on the number of floating cars in the queue, the arrival rate during the remaining red light and the subsequent changes in queue length will exhibit varying characteristics.

Case 1: When the number of floating cars in the queue is equal to 1

(1) By the time the floating car at the end of the queue enters the queue, the average arrival rate q of vehicles approaching the intersection during the red light period is:

$$q=\frac{L_{N}}{h}/\left(t_{N}^{*}-T_{1}\right)$$ (3)

Where *N* represents the ID of the tail floating car; $L_{N}$ indicates the distance between the floating car and the stop line; $t_{N}^{*}$ represents the time when the floating car at the tail of the queue enters the queue; h denotes the h-space headway; $T_{1}$ represents the start time of the red light.
(2) In reality, vehicle arrivals are random. Given the current sparsity of FCD data, if we assume the arrival rate remains constant throughout the entire red light phase, then the number of vehicles arriving during the remaining red light phase is:

$$Q=q\left(T_{2}-t_{N}^{*}\right)$$ (4)

Where $T_{2}$ denotes the end of the red light.
(3) The maximum queue length $L_{a}$ of the intersection is equal to the position of the floating car at the end of the queue plus the remaining red light time stage. The total length of the vehicles arriving at the end of the queue is:

$$L_{a}=L_{N}+Qh=\frac{L_{N}\left(T_{2}-T_{1}\right)}{t_{N}^{*}-T_{1}}$$ (5)

Since there is only one floating car in the queue, data from only this single floating car can be utilized, which is prone to significant estimation deviations. This makes it difficult to accurately reflect the actual situation of the entire transportation network. In the absence of other data sources for comparison and verification, this estimation bias may go undetected and uncorrected in a timely manner.

Case 2: When there are multiple floating cars in the queue (greater than 1),

First, identify the floating car positioned at the tail of the queue. Next, calculate its arrival rate relative to each downstream floating car. Given the continuity of traffic flow, the arrival rate of the floating car situated near the tail of the queue is considered to be more representative of the arrival rate during the remaining red light period. Therefore, this particular floating car’s data is assigned a higher weight in the calculation.

Utilizing the weighted average method, the weighted average arrival rate for the remaining red light period is computed, taking into account the weights assigned to each floating car based on their proximity to the tail. The term “new queue length” refers to the count of vehicles that have newly arrived during the red light period.

**Fig 5:**
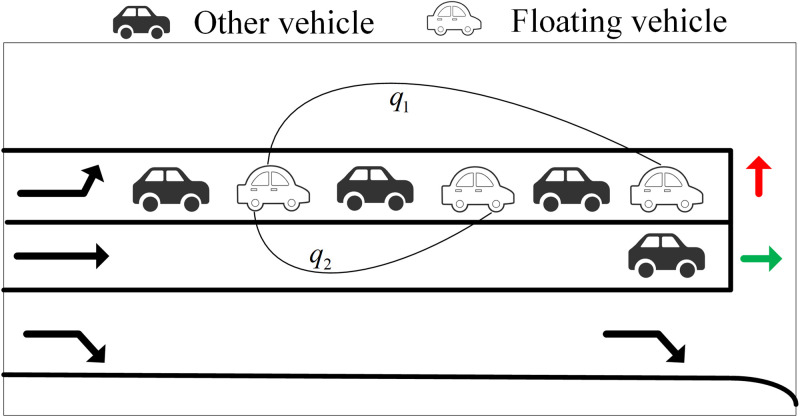
Schematic diagram of the calculation process of the remaining red light time arrival rate.

To estimate the maximum queue length, the new queue length is added to the position of the tail floating car within the queue. This calculation is visually depicted in Fig 5.

(1) Calculate the arrival rate $q_{i}$ for each floating car i downstream of the last floating car in the queue, relative to the last floating car in the queue:

$$q_{i}=\frac{L_{N}-L_{i}}{h\left(t_{N}^{*}-t_{i}^{*}\right)}$$ (6)

Where $L_{i}$ represents the distance between the i -th floating car and the stop line; $t_{i}^{*}$ represents the time when the i -th floating car enters the queue.
(2) Determine the weight corresponding to the arrival rate, which is proportional to the distance from the floating car at the tail of the team. Let $\omega_{i}$ be the weighting coefficient, then we can get:

$$\{\begin{array}{c} \omega_{i}=\frac{\mu_{i}}{\sum_{i=1}^{N-1}\mu_{i}}\\ \sum_{i=1}^{N-1}\omega_{i}=1\\ \mu_{i}=\frac{\text{1}}{L_{N}\;-\;L_{i}} \end{array}$$ (7)

Where *µ*_*i*_ denotes the reciprocal of the distance between the floating car at the end of the platoon and the floating car downstream.
(3) The maximum queue length $L_{a}$ of the intersection is equal to the product of the queue length of the floating car at the end of the queue plus the remaining red light time and its arrival rate:

$$L_{a}=L_{N}+h\left(T_{2}-t_{N}^{*}\right)\sum_{i=1}^{N-1}\omega_{i}q_{i}$$ (8)

## 3 Queueing vehicle number estimation

The following outlines the method for calculating the number of queuing vehicles $N_{a}\left(k\right)$, based on the real-time queuing length $L_{a}\left(k\right)$. At unsaturated signalized intersections, vehicles start to queue up during red lights, and their speed within the queue is minimal or zero. Consequently, the number of queuing vehicles can be determined by utilizing the average spacing between the queued vehicles.


$$N_{a}\left(k\right)=\frac{{\hat{L}}_{a}\left(k\right)}{h}$$ (9)

Where ${\hat{L}}_{a}\left(k\right)$ is determined by Eq (8).

The Equation mentioned earlier primarily applies when vehicle speeds within the platoon are nearly zero. However, in real-world traffic scenarios, traffic flow regulated by signals exhibits complex dynamic behaviors. Specifically, at unsaturated signalized intersections, vehicles downstream may not always form a static queue. When the signal turns green, the rear vehicles may start moving instead of remaining stationary. Therefore, the aforementioned Equation fails to account for these dynamic changes and may not accurately estimate the number of queuing vehicles, making it suitable only for relatively stable traffic conditions.

As traffic conditions evolve, including changes in signal cycles, flow fluctuations, and driver behavior uncertainties, more sophisticated and dynamic models are necessary to capture the nonlinearity and instability of traffic flow. Floating car data, which provides both vehicle position and detailed speed information, is crucial for accurately estimating the number of queuing vehicles $N_{a}\left(k\right)$. By employing a nonlinear function outlined in Eq (8), we can obtain an estimated value for ${\hat{N}}_{a}\left(k\right)$.


$${\hat{N}}_{a}\left(k\right)=f\left(N_{a}\left(k\right),V_{a}\left(k\right),\beta \right)$$ (10)

Where $V_{a}\left(k\right)$ represents the average speed (km/h) of the floating car in the team; β represents the unknown parameter vector; f(·) represents a data fusion function based on the Kalman filter.

According to the Eq (10), using the estimated value of ${\hat{L}}_{a}\left(k\right)$ and the assumed value of h, it can be deduced that the values of $N_{a}\left(k\right)$ and h are different with the change of traffic scene. Therefore, it is more appropriate to directly use ${\hat{L}}_{a}\left(k\right)$ as the input parameter for the nonlinear function in Eq (11):


$${\hat{N}}_{a}\left(k\right)=f\left({\hat{L}}_{a}\left(k\right),V_{a}\left(k\right),\beta \right)$$ (11)

The nonlinear function (11) takes into account both the speed and spacing of vehicles to reflect the complexity of real-world traffic flow. Assuming a linear relationship exists between the speed $V_{i}$ of each vehicle and its inter-vehicle spacing $H_{i}$, denoted as $V_{i}=-A+BH_{i}$, when $V_{i}=0$, $H_{i}=A/B=h$, and known parameter h, there is only one degree of freedom between parameters A and B. From this, we can derive the average speed $V_{a}$ of all floating cars queued downstream of the tail of the traffic jam.


$$V_{a}=-A+BH_{a}$$ (12)

Where $H_{a}$ denotes the average distance (km) between vehicles downstream of the platoon.

Both sides of Eq (12) are multiplied by the vehicle density $K_{a}$:


$$K_{a}V_{a}=-AK_{a}+BH_{a}K_{a}$$ (13)

And because.


$$K_{a}V_{a}=Q_{a},H_{a}K_{a}=1$$ (14)

The flow-density relationship is obtained:


$$Q_{a}=-AK_{a}+B$$ (15)

Where −A represents the negative slope of the triangular flow-density fundamental diagram, usually called the dissipation rate, which is usually-4m/ s.

Replacing the original h in Eq (9) with $H_{a}$, based on Eq (12) and relationship B=A/h, and through the derivation of Eq (16) and (17), the nonlinear function (18) is finally obtained:


$$N_{a}\left(k\right)=\frac{{\hat{L}}_{a}\left(k\right)}{H_{a}}$$ (16)

$$\frac{\text{1}}{H_{a}}=\frac{B}{V_{a}+A}=\frac{A}{h(V_{a}+A)}$$ (17)

$${\hat{N}}_{a}=\frac{A{\hat{L}}_{a}}{h\left(V_{a}+A\right)}$$ (18)

Eq (18) involves two unknown parameters, A and h, whose values can be determined by solving the following least squares problem using dataset $\left\{{\hat{N}}_{a}\left(k\right),{\hat{L}}_{a}\left(k\right)\right\}$:


$$\hat{\theta }=argmin_{\theta }\sum_{k=1}^{K}{\left[{\hat{N}}_{a}\left(k\right)-f\left({\hat{L}}_{a}\left(k\right),V_{a}\left(k\right),\theta \right)\right]}^{2}$$ (19)

Where f is the right side of Eq (18), θ={A,h}.

The unknown parameters of the model are determined through the calibration process. A and h represent the physical characteristics of actual traffic flow and, even without meticulous adjustment, can be reasonably approximated with assumed values. Consequently, the model can be utilized to calculate the number of queuing vehicles without the need for prior calibration.

## 4 Macroscopic fundamental diagram acquisition

To estimate the total number of vehicles operating within a specific area, this paper employs a method that relies on the maximum queue length of a road segment when only floating car data is available. The proportional coefficient is defined as the ratio of the number of queuing vehicles to the total number of queuing floating vehicles. This coefficient indicates the percentage of floating vehicles among all vehicles in motion and serves as a crucial parameter for estimating the overall vehicle count in the region.


$$p=\sum_{a=1}^{n}\overset{̑}{N_{a}}(k)/\sum_{a=1}^{n}N_{Fa}(k)$$ (20)

Where p represents the proportional coefficient; $N_{Fa}\left(k\right)$ denotes the total number of floating car queues (veh) on road *a*; ${\hat{N}}_{a}\left(k\right)$ denotes the number of vehicles in line (veh) on section a.

Based on the proportion coefficient and the total number of floating cars, the total number of running vehicles in the region is estimated in Eq (21).


$$N(k)=N_{F}(k)\cdot \;p=N_{F}(k)\cdot \sum_{a=1}^{n}{\hat{N}}_{a}(k)/\sum_{a=1}^{n}N_{Fa}(k)$$ (21)

Where *N*_*F*_(*k*) represents the total number of regional floating cars (veh); N(k) represents the total number of vehicles running in the region (veh).

Using Eqs (20) and (21), one can estimate the total number of vehicles operating in a specific area during different time periods. By incorporating the road area or length of the region, the vehicle density within that area can be determined. In traffic flow theory, vehicle density is typically closely linked to two other crucial parameters: vehicle speed and traffic flow. The Equation for this calculation is provided below:


$$Q=\rho \times V_{L}$$ (22)

$$V_{L}=\sum_{i=1}^{n}b_{i}V_{Li}/n$$ (23)

Where Q represents the average flow (veh/h); ρ represents the average density (veh/km); *V*_*L*_ represents the average travel speed (km/h); $b_{i}$ represents the weight coefficient of the road section, $0\leq b_{i}<1$ and $\sum b_{i}=1$. In this paper, according to the expressway: main road: secondary road: branch = 0.4: 0.3: 0.2: 0.1 to determine; $V_{Li}$ represents the travel speed of the road section (km/h); n represents the number of road sections.

## 5 Example analysis

### 5.1 Acquisition and preprocessing of floating car data

#### 5.1.1 Data description and preprocessing.

The data used in this paper mainly includes two parts: the first part is the road vector data of Chengdu city, and the second part is the GPS data set of Chengdu taxi in referring to S1 Table. The traffic flow data used in this study was obtained from the Gaia Data Open Platform of Chengdu. This open-access dataset is published under a Data Sharing Agreement that permits academic use with proper attribution.

The road network data for Chengdu originates from the OpenStreetMap (OSM) database, utilizing the WGS1984 geographic coordinate system. Leveraging the OSMnx library [34], a Python package featured in Geoff Boeing’s blog, the Chengdu road network data is retrieved. The OSMnx library is employed to rectify the topology of the road network and enhance the efficiency of data processing tasks.

**Fig 6:**
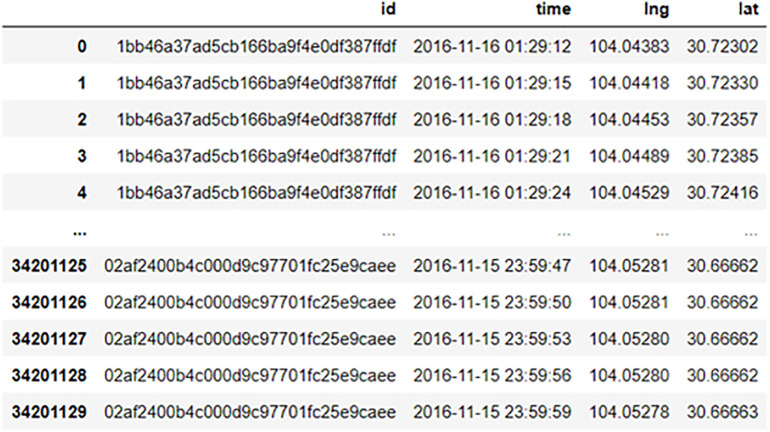
Example of floating car data.

The floating car data utilized in this paper is sourced from the Didi Chuxing Gaia Data Open Plan [35]. Specifically, data from November 16 to November 22, 2016, within the first to third rings of Chengdu, China (longitude range: 30.59–30.66, latitude range: 104.07–104.16) were selected for analysis. The original data disorderly stores the daily floating car data in a CSV file, with no temporal or ownership correlation between adjacent data points, necessitating subsequent processing. Fig 6 displays a sample of the data after being sorted by time.

#### 5.1.2 Floating car data content expansion.

The floating car data from the Gaia open platform contains only basic fields, such as vehicle ID, timestamp, GPS longitude, and GPS latitude. To derive various traffic operation parameters from these data, it is necessary to expand the existing data. The trajectory of a vehicle with the same order ID can be represented as follows:


$$Traj=\left\{{\left(id,t_{i},x_{i},y_{i}\right)}_{j}^{n}\right\}$$ (24)

Where *id* represents the order number; $t_{i}$ denotes the recording time of the i -th trajectory point; $x_{i}$ and $y_{i}$ represent the latitude and longitude coordinates of the *i*-th trajectory point; j denotes the position number of the trajectory point; n denotes the total number of all points in the trajectory.

After calculating the speed *ν*_*i*_, direction $dri_{i}$ and steering $tur_{i}$ of the trajectory point, the trajectory of the vehicle can be expressed as:


$$Traj=\left\{{\left(id,t_{i},x_{i},y_{i},dri_{i},v_{i},tur_{i}\right)}_{j}^{n}\right\}$$ (25)

Where $dri_{i}$ represents the forward direction azimuth of the trajectory point i, that is, the angle between the direction of the vehicle at the point and the north direction; $tur_{i}$ represents the steering angle of the trajectory point i compared to the forward direction of the previous point, reflecting the steering change of the vehicle between the continuous trajectory points.

#### 5.1.3 Map matching based on HMM-CRFs.

**Fig 7:**
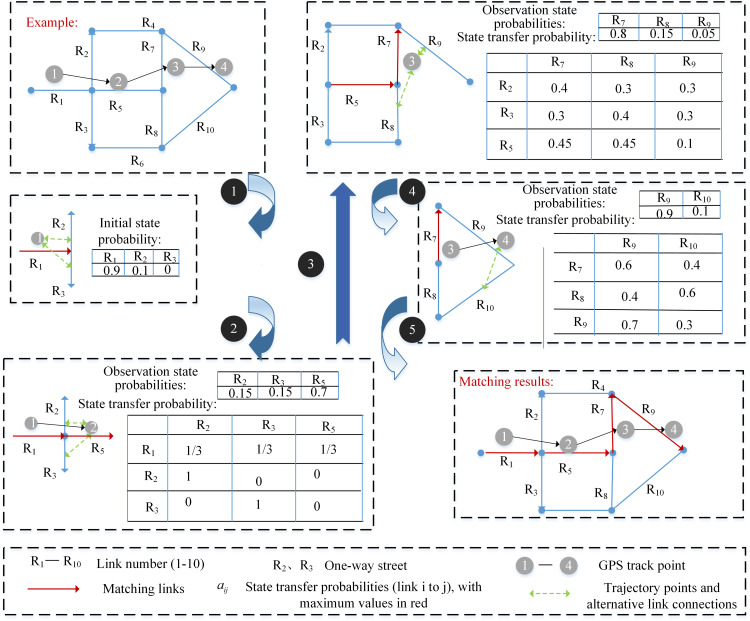
Example of a hidden Markov model.

Compared to earlier geometric, topological, and probabilistic algorithms, the map matching algorithm based on Hidden Markov Models (HMM) [36] demonstrates greater robustness and accuracy in practical applications. This algorithm considers each trajectory point as an observation point and the actual location of the taxi as a hidden state. It obtains the road network matching result by solving the HMM decoding problem. The implementation process is illustrated in Fig 7.

Although HMM has achieved notable results in map matching, there are still challenges and opportunities for improvement, one of which is the “label bias” problem. The “label bias” problem was first introduced by Lafferty et al. [37] One of the core assumptions of HMM is the Markov property, which states that the current state only depends on the previous state, and all observations are mutually independent. However, in the context of map matching, this assumption does not always apply. The driving path of a vehicle is typically not random but constrained by the road network structure and traffic regulations. The driving segment of a vehicle is not solely related to the preceding road but may also be influenced by various factors such as subsequent road segments, intersection traffic conditions, vehicle speed, and direction. HMM only considers the transition probability between adjacent road segments, neglecting these more intricate influencing factors.

When handling GPS trajectory data with a low sampling rate, the “label offset” problem becomes particularly evident. The HMM model may have a tendency to match GPS points to road segments that are longer and have fewer intersections. To mitigate the “label offset” problem, the HMM model can be enhanced by incorporating more contextual information, such as road type, traffic flow, and vehicle speed. Furthermore, more sophisticated probabilistic graphical models can be employed. Conditional Random Fields (CRFs) are defined within the framework of Markov processes and aim to more accurately label or predict sequences by considering the global features and contextual information of sequence data. CRFs can capture more complex dependencies between observations and hidden states compared to HMMs. By integrating additional features and considering the entire sequence context, CRFs can potentially provide more accurate map matching results, especially in scenarios with low-sampling-rate GPS data.

**Fig 8:**
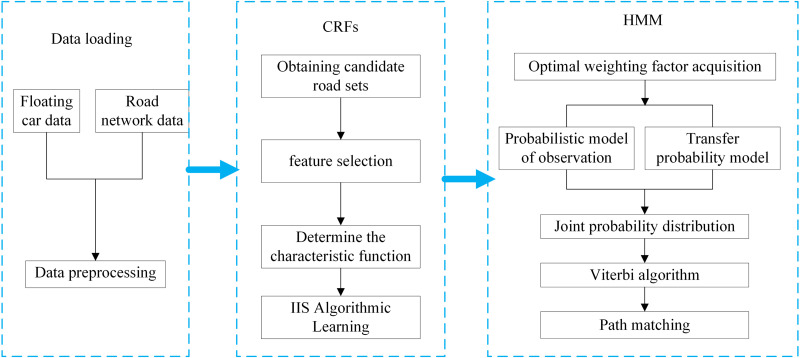
HMM-CRFs hybrid model framework.

Addressing the issues of the aforementioned HMM model, this paper introduces a hybrid model combining HMM and CRFs, as illustrated in Fig 8. The fundamental idea is to leverage the HMM model to capture local features of GPS trajectory points, such as the positional relationship between adjacent points and speed changes. Simultaneously, the CRFs model is employed to model global features and contextual information, encompassing aspects like road network structure, traffic regulations, and historical traffic data. By integrating these two models, the actual position of GPS trajectory points within the road network can be determined with greater accuracy. The process typically involves the following steps:

Step 1: Data Preprocessing: Preprocess GPS trajectory data and road network data to extract essential feature information.

Step 2: Candidate Road Segment Generation: Utilize the HMM model to generate a set of candidate road segments based on the local characteristics of GPS trajectory points. These segments represent potential roads that the GPS points could have traversed.

Step 3: Global Feature Modeling: Employ the CRFs model to capture global features and contextual information, constructing a conditional random field model for the entire road network.

Step 4: Posterior Probability Calculation: Combine the HMM model and CRFs model to compute the posterior probability of each candidate road segment. This probability indicates the likelihood that the GPS point actually traversed the segment.

Step 5: Optimal Path Matching: Apply the Viterbi algorithm to select the optimal matching path based on the posterior probabilities, determining the most probable GPS trajectory road sequence.

The algorithm code is given in Table 2.

**Table 2:** HMM-CRFs map matching code.

Algorithm: Hidden Markov Models-Conditional Random Fields, HMM-CRFs	
**Input:**	observations
**Output:**	states
**Steps:**	
1:	def generate_candidate_segments (preprocessed_gps, preprocessed_roads): #Candidate road section generation
2:	candidate_segments = [ ]
3:	for gps_point in preprocessed_gps:
4:	local_features = extract_local_features (gps_point, preprocessed_roads)
5:	segments = hmm_model.generate_candidates (local_features) #Suppose that the HMM model has been trained
6:	candidate_segments.extend (segments)
7:	return candidate_segments
8:	def model_global_features (preprocessed_roads): #Global feature modeling
9:	crf_model = train_crf_model (preprocessed_roads) #Assume that the CRFs model needs to be trained
10:	return crf_model
11:	def calculate_posterior_probabilities(candidate_segments, crf_model): #
12:	posterior_probs = {}
13:	for segment in candidate_segments:
14:	global_features = extract_global_features (segment, crf_model)
15:	posterior_probs[segment] = crf_model.calculate_posterior (global_features)
16:	return posterior_probs
17:	def select_optimal_path (candidate_segments, posterior_probs): #Optimal path matching
18:	optimal_path = viterbi_algorithm (candidate_segments, posterior_probs)
19:	return optimal_path

To validate the effectiveness of the proposed HMM-CRF hybrid algorithm, Table 3 systematically compares the performance of the standard Hidden Markov Model (HMM) and the HMM-CRF algorithm in terms of matching accuracy and computational efficiency.

**Table 3:** Performance comparison of map-matching algorithms.

Algorithm	Position Error (Mean ± SD, m)	Average Runtime (ms/point)	Average Candidate Links (links/point)
HMM Standard HMM	25.8 ± 18.5	12.5	5.2
HMM-CRF (Ours)	18.3 ± 12.1	16.8	5.2

Compared to the traditional HMM algorithm, the HMM-CRF algorithm proposed in this study significantly reduces the average position error by approximately 29%, while also markedly improving the standard deviation of the error. This demonstrates that the incorporation of the Conditional Random Field (CRF) not only effectively enhances the accuracy of map matching but also improves the stability of the matching results. Although the global inference mechanism of the CRF leads to an increase in algorithm runtime, it remains within an acceptable range, reflecting the practical value of the proposed method.

To quantitatively evaluate the contribution of the Conditional Random Field (CRF) component in the proposed hybrid model, we designed a systematic ablation experiment. Using the standard Hidden Markov Model (HMM) as the baseline, the experiment compares it with the complete HMM-CRF model across multiple key metrics. The results are presented in Table 4.

**Table 4:** Performance comparison of ablation study at intersection areas.

Evaluation Metric	HMM Standard HMM	HMM-CRF (Ours)	Performance Improvement
Overall Position Error (m)	25.8 ± 18.5	18.3 ± 12.1	Reduced by 29.1%
Position Error at Intersections (m)	35.6 ± 25.2	21.4 ± 15.8	Reduced by 39.9%
Intersection Matching Errors (count)	187	112	Reduced by 40.1%
Intersection Error Rate	12.3%	7.4%	Reduced by 39.8%
Average Runtime (ms/point)	12.5	16.8	Increased by 34.4%

The experimental results demonstrate that the incorporation of the CRF component significantly enhances model performance. In terms of matching accuracy, the positional error at intersections is reduced by 39.9% to 21.4 meters, while the mismatch rate decreases by 39.8% to 7.4%, proving the CRF’s effectiveness in resolving path selection ambiguity. Regarding global performance, the overall positional error is reduced by 29.1%, indicating the optimization’s comprehensive impact. As for computational efficiency, although the runtime increases by 34.4%, the processing speed of 16.8 ms per point still meets real-time requirements. In conclusion, by introducing spatiotemporal contextual constraints, the CRF component achieves an approximately 40% reduction in intersection mismatches at an acceptable computational cost, comprehensively improving matching accuracy and stability, thereby validating the effectiveness and practical value of the HMM-CRF hybrid architecture.

### 5.2 Macroscopic fundamental diagram acquisition

The study area’s traffic network is modeled as a topological graph consisting of 78 signalized intersections (nodes) and 129 roadway segments (edges). The network infrastructure includes 5 expressways, 28 primary arterial roads, and 24 secondary arterial roads, spanning a total of 36.3 kilometers. The trajectory data was gathered between November 1st and November 6th, 2016.

#### 5.2.1 Proportional coefficient calculation.

**Fig 9:**
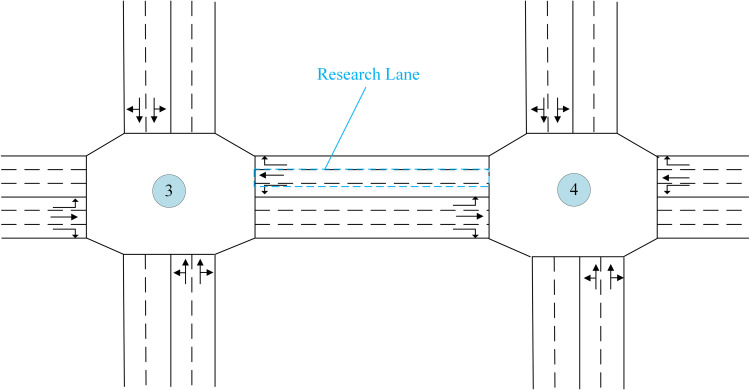
Study on the intersection layout diagram.

Due to space limitations, we will use the road segment between intersection 3 and intersection 4, traveling westward, as an illustrative example for analysis, as depicted in Fig 9. The processing methods for other road segments are identical.

**Fig 10:**
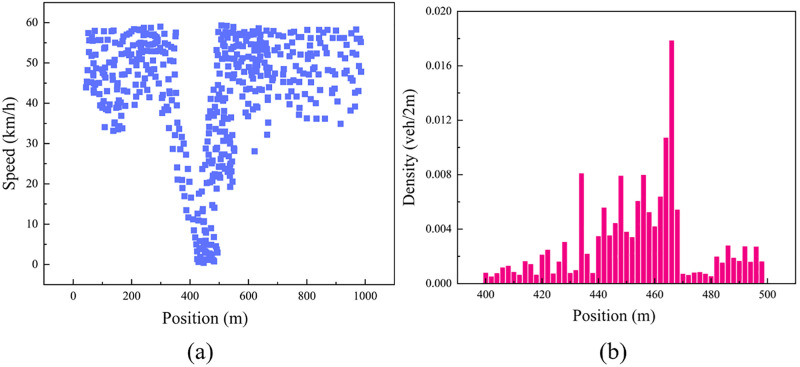
Parking line estimation: (a) the relationship between the speed and position of the floating car; (b) the density and position of the speed less than 5 km/h.

Subsequently, the relationship between vehicle speed and position was established, as shown in Fig 10(a). From this, it can be determined that the low-speed critical threshold $V_{th}$ is 5 km/h, and the intersection location range containing the stop line is identified as (400m, 500m). To further elucidate the characteristics of vehicle distribution, the selected floating car data is statistically analyzed at segments of 2 meters, yielding the vehicle density at each segment. The histogram in Fig 10(b) clearly depicts the relationship between density and location. Through thorough analysis and interpretation of these data, the precise location of the parking line is estimated to be at 595 meters.

The research time segment is 10 min, and the maximum queue length of the intersection is estimated according to Eq (8), as shown in Table 5.

**Table 5:** Maximum queue length.

Period	The distance between the floating car and the intersection (m)	The maximum queue length (m)
1	76.15	88.5
2	45.68	51.14
3	58.67	67.9
4	65.42	73.1
5	68.45	76.8
…	…	…

**Fig 11:**
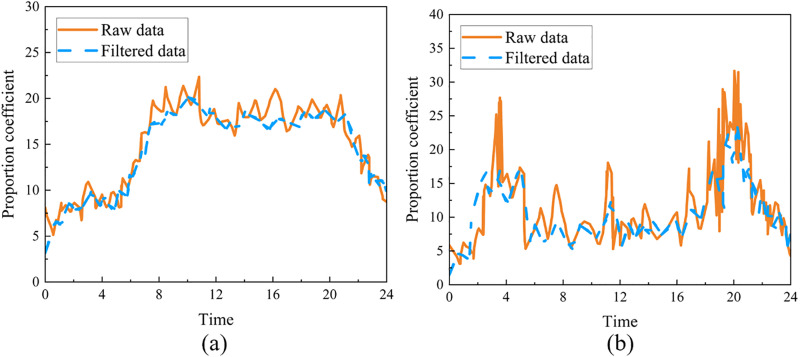
Proportional coefficient change curve: (a) working days. (b) non-working days.

The proportional coefficient derived from processing the floating car GPS data is presented in Fig 11. The coefficient’s curve exhibits significant fluctuations, aligning closely with the traffic flow trends. During peak hours, particularly in the morning and evening commutes, the coefficient tends to show a notable upward trend due to the substantial increase in traffic volume. Furthermore, on weekdays, the coefficient experiences larger fluctuations. Conversely, the average proportional coefficient tends to be higher on weekends compared to weekdays, highlighting the distinct characteristics of traffic flow distribution between these days.

To mitigate the impact of peak values on the calculation of the proportional coefficient, this paper employs data processed through a moving average filter to determine the average proportional coefficient.



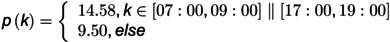

(26)


Where p(k) represents the ratio of the total number of vehicles operating within the specified area at time k to the number of floating vehicles recorded during that time.

The dynamic variation of the proportional coefficient is highly correlated with the number of private cars in the road network. During morning and evening peak hours, the surge in commuting demand leads to a significant increase in the number of private cars, resulting in a corresponding rise in the proportional coefficient. In contrast, during off-peak hours, the number of private cars remains relatively stable, and the fluctuation range of the proportional coefficient narrows significantly, with its value notably lower than during peak hours. This phenomenon confirms the overall stability of traffic flow during off-peak periods.

To quantify the representativeness bias of the taxi Floating Car Data (FCD) used in this study, we compared it with official data from the Chengdu Transportation Development Annual Report (2017). The report indicates that the average hourly traffic volume during weekday evening peak hours (17:00–18:00) in 2016 within Chengdu’s Third Ring Road area was approximately 1.25 million vehicle trips. By comparing the equivalent vehicle miles traveled estimated from the FCD data during the same period with the total vehicle miles traveled reported, this study calculated a global effective penetration rate of approximately 9.5%. This result confirms that taxis constitute a limited proportion of the overall traffic flow. Further regional analysis revealed significant spatial heterogeneity: in central urban areas (e.g., within the Second Ring Road), the penetration rate can reach 14.58%, while in peripheral areas (e.g., between the Third Ring Road and the Ring Expressway), the penetration rate drops sharply to 3.2%. This severe spatial imbalance leads to systematic deviations when directly using raw FCD data to estimate the total number of vehicles across the network, specifically manifesting as an overestimation of traffic density in central areas and an underestimation in peripheral areas.

#### 5.2.2 Acquisition of the macroscopic fundamental diagram.

**Fig 12:**
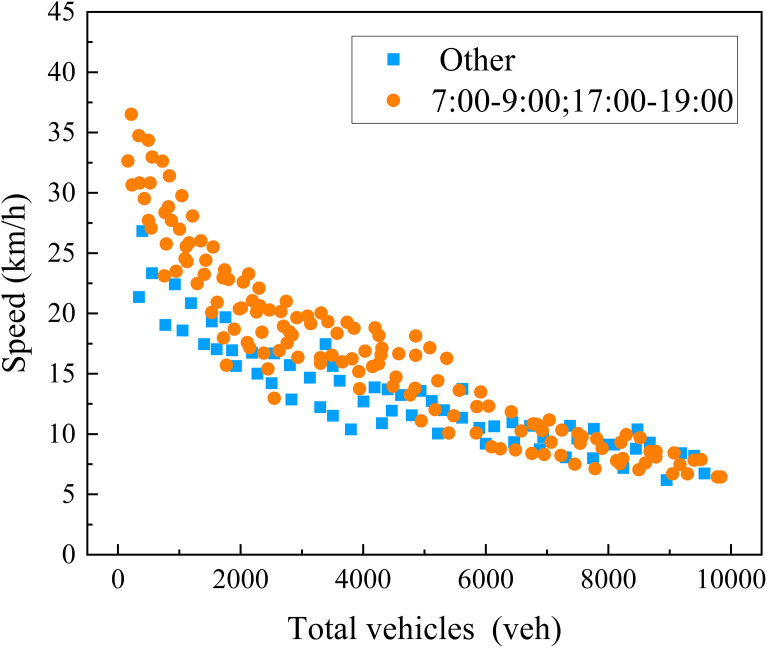
Macroscopic fundamental diagram of total number of vehicles and speed.

Fig 12 illustrates the dynamic interplay between the average speed of vehicles and the total number of vehicles within the region across various timeframes. When the total number of vehicles in the area is low, the average speed tends to be higher, as vehicles have minimal interaction with each other, resulting in smoother road traffic. However, as the number of vehicles increases, the level of road congestion gradually rises, leading to a corresponding decrease in the average speed of vehicles. This declining trend underscores the adverse effect of traffic congestion on driving efficiency.

**Fig 13:**
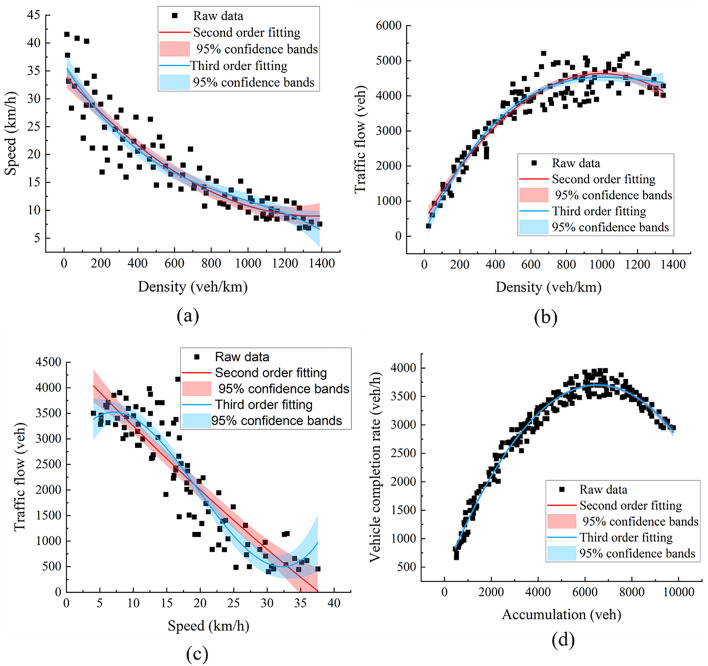
Region MFD drawing: (a) Vehicle density and speed; (b) Vehicle density and traffic flow; (c) Traffic flow and speed; (d)Accumulation Vehicle completion rate.

The fitting diagrams presented in Fig 13(a)–(d) illustrate the variation patterns of Density versus Velocity, Density versus Flow Rate, Traffic Flow versus Speed, and Accumulation versus Vehicle Completion Rate. The traffic flow in the road network increases as the vehicle density within the network rises. However, the rate of flow growth slows down when the density reaches a certain critical value, at which point congestion begins to emerge in the road network. Meanwhile, the average travel speed of vehicles within the network shows a similar trend, decreasing as the flow increases. This indicates that the traffic flow data processed based on floating car data align with the density-velocity and flow-density relationships in traffic flow theory, further validating the reliability of the macroscopic fundamental diagram obtained through the data processing method used in this section.

From Fig 13, it is evident that the regional Macroscopic Fundamental Diagram (MFD) exhibits a parabolic curve relationship. To analyze this relationship in detail, unary quadratic and unary cubic functions are selected to fit the scatter plot. The results of these fits are then evaluated using specific goodness-of-fit indicators listed in Table 4. These indicators include the Coefficient of Determination (R-squared), the Root Mean Square Error (RMSE), and the p-value of the F-test comparing the cubic model against the quadratic model. The R-squared value measures the extent to which the model explains the data, ranging from 0 to 1. Values closer to 1 indicate a better model fit. Conversely, RMSE assesses the deviation between observed and actual values. To statistically determine whether the added complexity of the cubic model is justified, an F-test was conducted for each MFD relationship. This test evaluates if the cubic term leads to a statistically significant reduction in the residual sum of squares compared to the quadratic model.

As seen in Table 6, the unary cubic curve provides the best fit, effectively explaining the variable relationships within the MFD. Furthermore, the F-test results confirm that the improvement in fit offered by the cubic model over the quadratic model is statistically significant (p < 0.05) for all three MFD relationships. The fitting function for this basic diagram is provided below:

**Table 6:** Goodness of fit and model significance test.

MFD	Function	R^2^	RMSE	F-statistic	p-value
Density-velocity	Unary quadratic function	0.8566	20.8621	__	___
	Unary cubic function	0.8963	18.5807	12.45	<0.001
Density – flow rate	Unary quadratic function	0.8956	25.2462	___	___
	Unary cubic	0.9012	23.1769	5.82	0.017
Traffic flow-speed	Unary quadratic function	0.8493	19.5834	___	___
	Unary cubic	0.8621	17.1782	8.91	0.003
Accumulation-vehicle completion rate	Unary quadratic function	0.8724	15.3268	___	___
	Unary cubic	0.9157	12.0943	15.23	<0.001

Density-velocity: $y=2.1656\cdot 10^{-6}\cdot x^{3}-0.0086\cdot x^{2}+10.9204\cdot x+122.5636$

Density-traffic flow: $y=-4.8481\cdot 10^{-11}\cdot x^{3}+9.5880\cdot 10^{-7}\cdot x^{2}-0.0073\cdot x+31.7623$

Traffic flow-speed:


$$y=-1.5576\cdot 10^{-8}\cdot x^{3}+4.6592\cdot 10^{-5}\cdot x^{2}-0.0563\cdot x+37.2896$$

Accumulation-vehicle completion rate:


$$y=-2.1894\cdot 10^{-10}\cdot x^{3}-7.4645\cdot 10^{-5}\cdot x^{2}+1.0038\cdot x+45.7047$$

### 5.3 Validation and comparative analysis

#### 5.3.1 Comparison with theoretical macroscopic fundamental diagram models.

To evaluate the theoretical rationality of the empirical models adopted in this paper, three classical theoretical Macroscopic Fundamental Diagram (MFD) models were selected as benchmarks for comparison:

- Greenshields’ linear model, whose speed-density relationship is $v=v_{f}(1-k/k_{j})$, where $v_{f}$ is the free-flow speed and $k_{j}$ is the jam density.
- Underwood’s exponential model, whose speed-density relationship is $v=v_{f}\exp(-k/k_{c})$, where $k_{c}$ is the critical density.
- Daganzo’s triangular fundamental diagram, whose flow-density relationship is a piecewise linear function expressed as: $q=\left\{ \begin{array}{c} v_{f}k,k\leq k_{c}\\ w\left(k_{j}-k\right),k>k_{c} \end{array}\right.$, where w is the backward wave speed in congested conditions and $k_{c}$ is the critical density.

These theoretical models, along with the unary quadratic and unary cubic empirical models, were fitted to the observed data. A comprehensive comparison was conducted using subsequent statistical indicators, as shown in the Table 7:

**Table 7:** Comparison of goodness-of-fit and statistical criteria for MFD models (Density-velocity).

Model Category	Model Name	R²	RMSE	AlC	BIC
Theoretical Model	Greenshields (Linear)	0.8012	24.15	1205.3	1215.1
Theoretical Model	Underwood (Exponential)	0.8324	22.87	1189.7	1199.5
Theoretical Model	Daganzo (Triangular)	0.8455	21.50	1175.2	1185.0
Empirical Model	Unary Quadratic Functio	0.8566	20.86	1168.9	1178.7
Empirical Model	Unary Cubic Function	0.8963	18.58	1140.5	1155.2

As shown in Table 7, the fitting performance of different theoretical models and empirical models is compared. From the perspective of the Coefficient of Determination (R^2^) and Root Mean Square Error (RMSE), the unary cubic empirical model achieves the best fit to the observed data. To further statistically identify the optimal model, we calculated the Akaike Information Criterion (AIC) and the Bayesian Information Criterion (BIC). These two criteria measure the goodness-of-fit while penalizing model complexity (number of parameters), where lower values indicate a better model. The results show that the unary cubic model has the lowest AIC and BIC values among all candidate models, providing strong statistical support for selecting it as the best descriptive model.

#### 5.3.2 Comparative analysis with measured data.

To quantitatively evaluate the accuracy of the FCD-based MFD estimation method proposed in this paper, we constructed a verification platform based on the VISSIM microscopic traffic simulation environment. This platform can provide precise ground-truth data while generating controllable floating car data streams.

Queue detectors were deployed on every lane of each entrance approach to accurately record the maximum queue length within each signal cycle. Virtual detectors were installed at all entrances and exits of the road network to precisely measure the input and output traffic flows. Simultaneously, the global vehicle list in VISSIM was used to directly obtain the cumulative number of vehicles in the network at each simulation time step. The scatter plot of (cumulative vehicle count, network flow) generated from this data is regarded as the “ground-truth MFD.”

To validate the overall MFD accuracy and quantify improvements, we defined a baseline method that directly calculates the arithmetic mean speed of all FCD samples as the network average speed, then uses Greenshields’ model to derive density and flow. The comparison among ground-truth MFD, baseline method estimates, and our proposed method’s estimates under the same simulation data is shown in Table 8:

**Table 8:** Comparison of MFD key parameter estimation errors with baseline method.

Method	Maximum Network Flow Rate	Critical Accumulated Vehicles	MFD Overall Fitting RMSE (veh/h)
Ground Truth	2850 veh/h	125 veh	–
Proposed Method	2750 veh/h	119 veh	185
Estimation Error	−3.5%	−4.8%	–
Baseline Method	2350 veh/h	135 veh	420
Estimation Error	−17.5%	+8.0%	–S
Performance Improvement	Relative Accuracy Improved by 80%	Absolute Error Reduced by 3.2%	Accuracy Improved by 56%

Table 2 demonstrates the superior performance of our proposed method compared to the baseline approach across all key metrics. Specifically, for the maximum network flow rate, our method reduces the estimation error from −17.5% to just −3.5%, achieving an 80% relative improvement in accuracy. In estimating critical accumulated vehicles, the error decreases substantially from +8.0% to −4.8%. Furthermore, the overall accuracy shows significant enhancement, with the RMSE for complete MFD series fitting improving from 420 veh/h to 185 veh/h, representing a 56% increase in precision. These results consistently validate the effectiveness of our method in providing more accurate MFD estimations.

#### 5.3.3 Parameter sensitivity analysis.

To evaluate the sensitivity and robustness of our method to key parameters, we conducted systematic parameter sweep experiments focusing on two critical parameters: the critical speed threshold and the average inter-vehicle spacing. The performance metrics across reasonable value ranges for each parameter are shown in Table 9.

**Table 9:** Sensitivity analysis of key parameters.

Parameter	Value	Queue Length RMSE (m)	Max Flow Estimation Error (%)	Critical Density Estimation Error (%)	MFD Overall RMSE (veh/h)
Critical Speed Threshold (km/h)	5	25.8	−5.2	+6.1	210
	10 (Baseline)	22.8	−3.5	−4.8	185
	15	24.1	−4.1	−5.3	195
	20	28.9	−6.8	−7.5	235
Average Inter-vehicle Spacing (m)	6.0	26.5	−10.5	+12.3	250
	7.0 (Baseline)	22.8	−3.5	−4.8	185
	7.5	23.2	+1.8	−2.1	190
	8.0	24.0	+6.9	+0.5	205

Analysis of Table 9 leads to the following conclusions: Firstly, within the reasonable value ranges of the key parameters, the core output metrics of our method remain stable, demonstrating its good robustness. Specifically: (1) When the critical speed threshold is between 10−15 km/h, the errors for all metrics are at their lowest levels, indicating this range as the optimal choice; (2) The average inter-vehicle spacing has the most significant impact on flow estimation, but when it is between 7.0–7.5 meters, all errors are controlled at relatively low levels.

## 6 Conclusion

This study addresses issues such as the limited deployment of fixed-loop detectors and inaccurate assumptions about floating car penetration rates by proposing an innovative method for obtaining the MFD based on GPS trajectory data. The method first utilizes spatiotemporal data from floating cars to dynamically identify stop-line positions. By calculating the distance between floating cars and the stop-line, and combining the position of the tail of the queue with the length of remaining arriving vehicles, it infers the maximum queue length within a cycle. Building on this, the study establishes a nonlinear function model based on the average vehicle speed to accurately calculate the number of queuing vehicles by analyzing traffic flow characteristics within the queue area. Furthermore, a dynamic proportionality coefficient between private cars and floating cars is determined based on the maximum queue length of road segments. This coefficient is multiplied by the total number of floating cars in the area to achieve an assumption-free estimation of the total number of vehicles operating in the region.

To validate the effectiveness of the method, this study systematically processed and performed map-matching on Chengdu’s road vector data and taxi GPS datasets, successfully constructing a regional MFD. Through fitting analyses of the relationships between vehicle density and speed, density and flow, and the total number of operating vehicles and speed, it was found that the unary cubic curve significantly outperformed the unary quadratic function and classical theoretical models such as Greenshields, Underwood, and Daganzo’s triangular diagram in terms of the coefficient of determination, root mean square error, and AIC/BIC statistical criteria. Thus, it was identified as the optimal fitting form.

Simulation validation results demonstrate that the proposed method achieves an accuracy of RMSE 22.8 meters and MAPE 18.5% in estimating intersection queue lengths. In terms of MFD construction, compared to the baseline method, the estimation error for the maximum network flow rate was significantly reduced from −17.5% to −3.5%, representing an 80% relative improvement in accuracy. These quantitative findings fully confirm the effectiveness and superiority of the proposed method in enhancing the accuracy of MFD acquisition, providing a new technical approach for evaluating the operational status of urban road networks.

However, despite achieving certain research outcomes, this study has several limitations. First, the taxi-based dataset suffers from uneven spatial coverage and sampling bias in vehicle types, which may affect the accuracy of MFD estimates in specific sub-regions. Second, the current temporal resolution of floating car data (e.g., 2–3 minutes per point) limits its ability to capture instantaneous traffic dynamics. Finally, the validation results of the method in Chengdu cannot be directly generalized to cities with different road network structures or traffic regulations. To address these limitations, future work will explore the integration of multi-source data to improve data representativeness, develop adaptive algorithms to enhance model transferability, and deeply integrate advanced machine learning methods such as graph neural networks with traffic flow theory to improve the handling of spatiotemporally incomplete data.

## Supporting information

S1 Table Chengdu taxi data for November 19, 2016. (CSV)
